# Patient experience of access to primary care: identification of predictors in a national patient survey

**DOI:** 10.1186/1471-2296-11-61

**Published:** 2010-08-28

**Authors:** Evangelos Kontopantelis, Martin Roland, David Reeves

**Affiliations:** 1 National Primary Care Research and Development Centre, University of Manchester, Manchester, M13 9PL, UK; 2 General Practice and Primary Care Research Unit, University of Cambridge, Cambridge CB2 0SR, UK

## Abstract

**Background:**

The 2007/8 GP Access Survey in England measured experience with five dimensions of access: getting through on the phone to a practice, getting an early appointment, getting an advance appointment, making an appointment with a particular doctor, and surgery opening hours. Our aim was to identify predictors of patient satisfaction and experience with access to English primary care.

**Methods:**

8,307 English general practices were included in the survey (of 8,403 identified). 4,922,080 patients were randomly selected and contacted by post and 1,999,523 usable questionnaires were returned, a response rate of 40.6%. We used multi-level logistic regressions to identify patient, practice and regional predictors of patient satisfaction and experience.

**Results:**

After controlling for all other factors, younger people, and people of Asian ethnicity, working full time, or with long commuting times to work, reported the lowest levels of satisfaction and experience of access. For people in work, the ability to take time off work to visit the GP effectively eliminated the disadvantage in access. The ethnic mix of the local area had an impact on a patient's reported satisfaction and experience over and above the patient's own ethnic identity. However, area deprivation had only low associations with patient ratings. Responses from patients in small practices were more positive for all aspects of access with the exception of satisfaction with practice opening hours. Positive reports of access to care were associated with higher scores on the Quality and Outcomes Framework and with slightly lower rates of emergency admission. Respondents in London were the least satisfied and had the worst experiences on almost all dimensions of access.

**Conclusions:**

This study identifies a number of patient groups with lower satisfaction, and poorer experience, of gaining access to primary care. The finding that access is better in small practices is important given the increasing tendency for small practices to combine into larger units. Consideration needs to be given to ways of retaining these and other benefits of small practice size when primary care services are reconfigured. Differences between population groups (e.g. younger people, ethnic minorities) may be due to differences in actual care received or different response tendencies of different groups. Further analysis is needed to determine whether case-mix adjustment is required when comparing practices serving different populations.

## Background

Access to health services is a prerequisite for any high quality health care system. Conceptually, access can be classified as a dimension of care on its own, separated from dimensions of quality [1,2] though it has more often been seen as one of the essential elements of quality [3,4]. For the National Health Service (NHS), access is a high policy priority. The NHS Plan [5] in 2002 stated that patients should be able to see a health professional within 24 hours and a general practitioner within 48 hours, and in 2004, GPs were given a financial incentive to achieve this target Many GPs responded to the incentive by using a model of 'Advanced Access' which attempts to match demand and capacity on a day-to-day basis [6]. In the US, this model has been successful in both accelerating entry into the system and reducing the strain on clinical resources [7]. However, many UK practices also substantially reduced the number of appointments that could be booked in advance in an attempt to meet the 24/48-hour target. This reduced patient's ability to book ahead, and may have been one factor associated with a reduction in continuity of care [8], since patients found it harder to be seen by their preferred doctor on the day of their choice [9].

The possible deterioration in patient access to primary care led the Department of Health to create the Improved Access Scheme, in an effort to evaluate and further improve access by incentivising the ability to book ahead as well as the ability to get appointments rapidly [10]. The scheme used a patient survey to evaluate access, and since 2006, an annual survey has been used to measure a range of dimensions of access to GP surgeries, and to reward practices for their performance.

The reports for the first two years of the survey indicated that positive experience and satisfaction was high in all five dimensions of access. However, the reports were limited to a descriptive exploration of the outcomes. In this paper, using data from the 2007/08 wave of the survey, we explore the factors associated with patient satisfaction and experience at the level of: (1) the patients; (2) the practices; and (3) the geographical region.

## Methods

### GP Access survey

The survey was administered by Ipsos MORI, on behalf of the Department of Health. Patient information was obtained using Primary Care Trust (PCT) registration records from the National Health Application and Infrastructure Services (NHAIS) database. The main outcomes of interest were the survey items that asked respondents about their experience and satisfaction with access to their general practice. Satisfaction and experience with access are two theoretically different aspects of care [11]. The questionnaire included two items relating to satisfaction with aspects of access (Q2, satisfaction with how easy it is to get through to someone on the phone at the surgery; and Q9, satisfaction with the hours the surgery was open) and three items relating to experience with aspects of access (Q4, experience with getting an appointment on the same day or on the next 2 days the surgery was open; Q6, experience with making an appointment with the surgery more than 2 full days in advance; and Q8, experience with making an appointment with a particular doctor at surgery even if it meant waiting for longer - all restricted to patients who had tried in the preceding six months). All five items were dichotomous and answered with a 'Yes' or 'No'. The five items were agreed between the British Medical Association and NHS employers. Ipsos MORI undertook a series of face to face cognitive tests to examine if the questions were clear and easily understood. Some limitations were identified and the questionnaire was redesigned after each round of testing. After the first year of the survey, some changes were made to the questionnaire which did not compromise its consistency [12].

Information on patient characteristics was also collected in the questionnaire: age, gender, number of appointments in the last year, whether the patient was a parent or legal guardian, employment status, travel time from home to work, typical working hours, ability to take time away from work to visit GP surgery, limiting long-standing conditions, difficulty performing day-to-day activities because of limiting long-standing conditions, carer responsibilities and ethnicity. Measures of area deprivation and rurality were assigned to patients based on the Lower Super Output Area in which each resided. The full 2008 questionnaire is presented in Additional file 1.

In 2007/08, 8,307 out of 8,403 practices in England were included in the survey (reasons for exclusion included having fewer than 50 eligible patients). Patients were eligible to be selected for participation if they were aged 18 or over, with a valid NHS number and registered with the same practice continuously from the 1 July 2007 to the date of the sample extraction from the NHAIS on 18/19 November 2007. Patients were randomly sampled from each participating practice, with more patients selected in practices likely to have lower rates of response. The sample size for each practice was determined by the number of returned questionnaires likely to deliver a confidence interval of ± 7 percentage points, at the 95% level, for items Q2, Q3 and Q4 [12]. The questionnaires and a cover letter were posted in the week commencing on the 7^th ^of January 2008, with two reminders sent out in February and March, while the closing date for completed surveys was the 2^nd ^of April 2008. Overall 4,922,080 questionnaires were posted, with no more than 930 issued for any practice. Telephone help lines in 10 languages in addition to English were available for individuals who were unable to complete the questionnaire without additional assistance. The overall response rate was 40.6%, with 1,999,523 completed responses collected [13]. More details on the development and organisation of the survey can be obtained from the technical report published by Ipsos MORI [12]. The dataset is not publicly available.

For this study, we obtained information about practice and PCT characteristics from other additional sources: the General Medical Services (GMS) database 2006; Super Output Area Indices of Multiple Deprivation 2004; and the Quality and Outcomes Framework (QOF) results for 2006/7. Practice level variables were: practice list size, full time equivalent GPs, ratio of full time equivalent GPs per 10,000 patients, overall reported achievement on 48 'stable' QOF indicators (i.e. introduced in 2004/05 and with minor or no changes in the first 5 years of the scheme), distance to nearest practice, emergency admissions per 1000 patients, standardised mortality ratios of people under 65, number of new registrations, total opening hours and extended opening hours. Measures of global practice population deprivation and rurality were created by aggregating scores across the patients in each practice sample. We constructed practice population measures of ethnic mix, percentage of people in full-time employment and age profile, using both the practice samples and the 2001 census. Both estimates are prone to error (those from the sample due to self-selection bias; those from the Census due to changes since 2001), however, the two measures correlated well for ethnic mix (White v non-White; r = 0.856) moderately well for age (mean age, r = 0.614), but less well for rates of full-time employment (r = 0.537). In the analysis we used to estimates from the Census. Regional information was limited to three variables: Strategic Health Authority, number of practice staff in the PCT per 100,000 population, and walk-in centre attendance in the PCT per 100,000 population (walk in centres existed in 49 of the 152 PCTs) and had been established specifically to improve access to primary care.

### Statistical Analyses

We used multilevel multivariate regression to investigate relationships between each dimension of satisfaction/experience and patient, practice and regional characteristics. The outcome variables were all binary (e.g. able/unable to get an urgent appointment), therefore we utilised logistic regression. We began with univariate analyses, examining each predictor separately, and followed these up with a multivariate analysis to control for relationships between predictors. We included the patient, practice and regional level predictor variables in the same multi-level analysis. The size of the dataset made it not feasible to model the full hierarchical nature of the data (respondents nested within practices nested within regions), therefore we adopted a two-level model that took account of the nesting of respondents within practices, and assigned the regional variables to the individual practices. Although this may have introduced some small error into the p-values for some predictors, p-values have not been used to gauge the importance of each predictor.

The size of the sample was such that very small differences in scores were statistically significant, making significance alone a poor guide to the effect of each predictor. Therefore to assess strength of effect we used an approach based on the odds-ratio coefficient for each predictor variable. First, we rescaled each continuous predictor variable by subtracting the mean and dividing by twice the variable's standard deviation. In analysis, these rescaled variables then yield odds-ratios comparable to those obtained comparing one level of a categorical variable with another [14]. Second, we defined an important predictor to be one with a calculated odds-ratio of 1.18 or above, or 0.86 or below. These values correspond to an increase/decrease in the satisfaction/experience score of 2.5% or more, from a baseline of 80% (the average across all five domains of satisfaction/experience).

We conducted one multivariate analysis on the full sample of patients, and a second using only those patients in full- or part-time employment. Three variables that were only applicable to patients in employment (travel-time from home to work, typical working hours and ability to take time away from work to visit GP surgery) were included in the second analysis only.

We excluded patients with any missing data, since the sample size was large enough to allow us to avoid less robust approaches. We examined the set of independent variables for multi-collinearity prior to analyses and removed those with a variance inflation factor greater than four [15]. One variable, full time equivalent GPs was removed due to multi-collinearity: this was highly correlated with practice list size and the latter was a stronger predictor in the univariate analyses. Total practice opening hours and extended opening were only available for around half of the practices (53%) and were not included in the presented regressions. However, we repeated the analyses including these variables and using only those practices for which we had data; their effect on the access items was not found to be important. All analyses were undertaken using STATA version 10.1 [16].

## Results

Table 1 displays the overall satisfaction/reported experience frequencies and scores. Table 2 displays the raw scores of satisfaction and reported experience for each predictor. Standardised odds ratios from the multivariate analyses, for predictors with a substantial relationship to domain scores in the main and sub-analysis, are shown in Tables 3 and 4 respectively. Table 5 gives percentages of practice-level variability explained by the patient- and practice-level predictor variables.

**Table 1:** Overall satisfaction/positive experience frequencies and scores.

	Access domains				
	
	Q2	Q4	Q6	Q8	Q9
	
	Satisfied with getting through on the phone	Able to get appointment same day or next 2 days	Able to get appointment >2 days in advance	Able to get appointment with particular GP	Satisfied with hours GP surgery open
No	246,953	164,823	212,522	128,701	343,526

Yes	1,708,294	1,028,478	671,516	840,943	1,602,492

Total	1,955,247	1,193,301	884,038	969,644	1,946,018

Percentage satisfied/able to get appointment	87.4%	86.2%	76.0%	86.7%	82.4%

**Table 2:** Raw scores of satisfaction and positive experience on the five access domains.

		Access domains				
		
		Q2	Q4	Q6	Q8	Q9
		
		Satisfied with getting through on the phone	Able to get appointment same day or next 2 days	Able to get appointment >2 days in advance	Able to get appointment with particular GP	Satisfied with hours GP surgery open
***Patient level predictors***		%	%	%	%	%

Gender	Male	87.8	86.0	77.9	86.8	82.3
	
	Female	87.1	86.3	74.8	86.7	82.4

Age group	18 - 34	83.0	81.5	69.7	79.7	75.4
	
	35 - 54	85.7	83.8	72.0	83.8	78.2
	
	55 - 74	89.2	88.4	78.9	89.5	86.0
	
	75+	91.7	92.2	85.1	91.5	90.6

Number of appointments (in last 12 months)	0 - 3	87.8	84.6	73.2	85.0	82.1
	
	4 - 6	86.9	86.9	77.3	87.9	81.9
	
	7+	86.7	88.6	78.9	88.0	84.0

Parent/legal guardian of any children under 16 in household	No	88.1	86.5	77.1	87.9	83.3
	
	Yes	84.5	85.0	71.6	82.3	78.4

Employment status	Full-time paid work	85.1	81.7	71.0	83.0	74.1
	
	Part-time paid work	86.9	86.7	73.9	86.1	82.8
	
	Full-time education	82.4	80.1	68.2	78.7	76.4
	
	Unemployed	85.7	85.0	74.6	81.0	86.7
	
	Perm sick/disabled	87.2	87.0	77.4	87.0	87.4
	
	Fully retired work	90.9	91.1	82.8	91.4	90.3
	
	Looking after home	87.7	88.6	77.2	87.3	86.0
	
	Something else	86.3	85.0	75.0	85.8	82.5

Travel time to work	Less than 10 min	87.3	86.2	74.3	85.9	82.1
	
	10-30 minutes	86.0	83.7	72.1	84.4	78.7
	
	31 minutes - 1 hour	84.1	80.3	70.0	82.1	70.0
	
	More than 1 hour	82.1	77.9	67.7	79.0	63.4
	
	Live on site	89.2	86.6	76.6	87.8	83.1

Typical working hours	Weekday office hrs	85.5	82.2	71.7	84.0	73.0
	
	Weekday mornings	87.2	87.2	74.7	85.9	85.8
	
	Weekday evenings or afternoons	85.4	85.6	72.2	83.1	83.7
	
	Overnights	85.1	84.3	72.8	83.1	81.6
	
	Weekends	84.0	82.4	68.3	81.4	78.9
	
	Other work pattern	85.5	83.3	71.4	83.7	78.8
	
	Working hours vary	85.7	83.3	71.6	83.9	78.5

Can take time away from work to visit GP	No	77.4	73.7	60.6	75.7	57.6
	
	Yes	88.6	86.5	76.6	87.2	82.7

Substantial difficulties in day-to-day activities because of long-standing hlth problem/disability	No	87.6	86.1	75.7	86.9	81.8
	
	Yes	86.9	87.0	77.5	87.5	85.7

Carer responsibilities for anyone in household with long-standing health problem or disability	No	87.5	86.0	76.0	86.8	82.2
	
	Yes	85.9	86.4	75.2	85.9	81.8

Ethnicity	White British	88.6	87.1	77.0	88.3	83.4
	
	Other white	85.8	83.5	74.6	84.6	79.0
	
	Black	83.3	83.6	72.4	78.5	81.1
	
	Asian	77.3	79.8	67.5	76.5	73.4
	
	Other	84.3	84.9	73.9	82.1	80.9

Deprivation† c(33) = 12.2 and c(66) = 24.9	Low	89.2	88.0	78.4	89.2	81.7
	
	Medium	87.8	86.5	76.3	87.3	82.2
	
	High	85.2	84.1	73.1	83.5	83.2

Rurality	Urban	86.4	85.3	74.8	85.7	82.2
	Rural	91.6	89.9	80.9	90.7	83.1

***Practice level predictors***		%	%	%	%	%

Contract type	PMS*	87.5	86.3	76.0	87.2	82.2
	
	GMS**	87.1	86.0	75.9	86.0	82.5

Practice list size	< 2,000	94.0	91.5	87.2	89.2	84.8
	
	≥2,000 & <6,000	89.6	86.8	78.9	88.1	82.7
	
	≥6,000 & <10,000	85.7	85.2	73.5	86.1	81.8

	≥10,000	83.0	84.9	71.1	84.8	81.6

Full Time Equivalent (FTE) GPs	1	90.2	87.1	80.5	86.8	82.7
	
	> 1 to 5	87.9	86.2	76.6	87.6	82.4
	
	5+	85.1	85.6	73.0	85.4	82.1

Full Time Equivalence ratio per 10,000 patients† c(33) = 5.1 and c(66) = 6.6	Low	86.0	84.4	74.8	85.6	81.6
	
	Medium	87.0	86.1	75.5	87.2	82.4
	
	High	89.0	87.8	77.4	87.2	83.1

Overall reported achievement (comparable indicators only) † c(33) = 89.4 & c(66) = 91.9	Low	85.2	84.1	73.2	85.0	81.5
	
	Medium	87.1	86.2	76.1	86.8	82.4
	
	High	89.7	88.2	78.6	88.4	83.1

Distance to nearest practice† c(33) = .223 & c(66) = .728	Low	86.8	85.4	75.3	86.2	82.6
	
	Medium	86.4	85.3	75.1	85.6	81.9
	
	High	88.9	87.8	77.5	88.3	82.6

Emergency admissions for patients on list per 1000 patients† c(33) = 69.1 & c(66) = 87.8	Low	89.8	87.7	79.9	88.9	81.3
	
	Medium	87.2	86.3	75.5	86.6	82.1
	
	High	85.2	84.7	72.4	84.5	83.6

GP referrals for patients on list per 1000 patients† c(33) = 714 & c(66) = 850	Low	89.5	87.9	78.9	88.6	82.2
	
	Medium	87.3	86.3	75.9	86.7	82.4
	
	High	85.4	84.5	73.1	84.8	82.4

Standardised Mortality Ratio, people under 65† c(33) = 88.3 & c(66) = 113.9	Low	89.9	88.7	79.5	89.3	81.6
	
	Medium	87.0	86.1	75.3	86.9	82.3
	
	High	85.4	84.0	72.9	83.7	83.1

Number of new † registrations c(33) = 343 & c(66) = 643	Low	91.0	88.5	81.1	89.2	84.1
	
	Medium	87.0	85.9	75.4	86.8	82.0
	
	High	84.2	84.3	72.3	84.7	81.0

Total hours a practice is open a week† c(33) = 30 & c(66) = 45	Below average	86.8	85.9	76.1	86.2	80.6
	
	Average	86.2	85.4	74.5	85.9	81.1
	
	Above average	87.8	86.5	76.3	87.1	83.2

Extended opening hours	No	87.0	85.9	75.9	86.5	81.7
	
	Yes	87.6	86.4	76.0	86.9	82.7

Practice Index of Multiple Deprivation score (aggregated from patient sample) † c(33) = 15.6 & c(66) = 26.7	Low	89.9	88.7	79.4	89.4	82.0
	
	Medium	87.1	86.2	75.4	86.9	82.3
	
	High	85.2	83.8	72.9	83.6	82.8

Practice rurality (% of sample patients living in a rural setting) † c(33) = 0.4% & c(66) = 7.7%	Low	84.6	82.9	73.8	83.7	80.2
	
	Medium	87.2	86.6	75.2	86.7	83.5
	
	High	90.2	89.0	78.7	89.4	83.3

*Region predictors (practice-level)*		%	%	%	%	%

Practice staff per 100,000 population† c(33) = 43.0 & c(66) = 73.5	Low	88.3	87.6	76.1	87.7	83.1
	
	Medium	87.7	86.9	76.5	87.1	83.1
	
	High	86.1	84.2	75.3	85.4	80.9

Walk in centre available in PCT	No	87.7	86.7	76.2	87.1	82.5
	
	Yes	86.7	85.1	75.4	85.9	82.1

Strategic Health Authority	North East	89.6	87.2	78.3	87.7	86.3
	
	North West	87.7	86.1	74.4	86.7	84.7
	
	Yorkshire/Humber	87.2	86.3	76.0	86.9	84.3
	
	East Midlands	86.6	87.3	73.3	86.2	82.9
	
	West Midlands	86.9	86.4	76.3	86.3	82.9
	
	East Of England	87.4	87.1	75.7	87.3	81.9
	
	London	84.2	81.7	74.6	83.1	77.5
	
	South East Coast	88.0	88.6	75.3	87.9	80.6
	
	South Central	89.7	87.5	79.2	88.9	81.8
	
	South West	90.9	89.2	79.9	89.9	84.4

* Personal Medical Services contract: an alternative contract offered by local authorities.

** General Medical Services contract: the standard contract under which practices are rewarded (65% of English practices)

† Continuous variables have been categorised using 33^rd ^and 66^tth ^percentiles for the purpose of this table

Q2: all predictors significant at the 99.9% level (p ≤ 0.001) except: Substantial difficulties in day-to-day activities because of long-standing health problem/disability (p = 0.460), deprivation (p = 0.002), contract type (p = 0.093), total hours a practice is open a week (p = 0.776), extended opening hours (p = 0.092).

Q4: all predictors significant at the 99.9% level (p ≤ 0.001) except: deprivation (p = 0.282), contract type (p = 0.037), total hours a practice is open a week (p = 0.938), extended opening hours (p = 0.877).

Q6: all predictors significant at the 99.9% level (p ≤ 0.001) except: carer responsibilities for anyone in household with long-standing health problem or disability (p = 0.988), deprivation (p = 0.053), contract type (p = 0.526), total hours a practice is open a week (p = 0.023), extended opening hours (p = 0.093).

Q8: all predictors significant at the 99.9% level (p ≤ 0.001) except: gender (p = 0.013), carer responsibilities for anyone in household with long-standing health problem or disability (p = 0.072), contract type (p = 0.526), total hours a practice is open a week (p = 0.011), extended opening hours (p = 0.744).

Q9: all predictors significant at the 99.9% level (p ≤ 0.001) except: gender (p = 0.008), rurality (p = 0.132), contract type (p = 0.526), distance to nearest practice (p = 0.005), GP referrals for patients on list per 1000 patients (p = 0.027), extended opening hours (p = 0.855).

**Table 3:** Associations between predictors and measures of patient satisfaction and experience, multilevel regression on all respondents.

		Access domains				
		
		Q2	Q4	Q6	Q8	Q9
		
		Satisfied with getting through on the phone	Able to get appointment same day or next 2 days	Able to get appointment >2 days in advance	Able to get appointment with particular GP	Satisfied with hours GP surgery open
***Number of patients in regressions***		1,612,203	981,587	733,390	804,561	1,611,139

***Number of practices in regressions***		8,038	8,038	8,038	8,038	8,038

***Patient level predictors***		**Standardised Odds Ratios**				

Gender	Male	-	-	-	-	-
	Female	***0.908	***0.969	***0.812	***0.969	***0.892

Age group	18 - 34	-	-	-	-	-

	35 - 54	***1.167	***1.094	***1.085	***1.276	***1.133

	55 - 74	***1.315	***1.337	***1.266	***1.599	***1.289

	75+	***1.677	***1.781	***1.744	***1.795	***1.483

Number of appointments (in last 12 months)	0 - 3	-	-	-	-	-

	4 - 6	***0.910	***1.157	***1.179	***1.271	***0.872

	7+	***0.910	***1.350	***1.333	***1.391	***0.921

Employment status	Full-time paid work	-	-	-	-	-
	
	Part-time paid work	***1.198	***1.370	***1.175	***1.245	***1.746
	
	Full-time education	***1.068	***1.097	0.963	**1.083	***1.381
	
	Unemployed	***1.453	***1.506	***1.360	***1.176	***2.665
	
	Perm sick/disabled	***1.588	***1.753	***1.535	***1.512	***2.885
	
	Fully retired work	***1.580	***1.909	***1.610	***1.718	***2.945
	
	Looking after home	***1.405	***1.653	***1.435	***1.457	***2.401
	
	Something else	***1.168	***1.221	***1.223	***1.266	***1.802

Substantial difficulties in day-to-day activities because of long-standing health problem/disability	No	-	-	-	-	-

	Yes	***0.781	***0.778	***0.826	***0.813	***0.805

Ethnicity	White British	-	-	-	-	-

	Other white	***0.848	***0.884	***0.867	***0.801	***0.846

	Black	***0.908	***1.158	**0.947	***0.709	***1.134

	Asian	***0.701	***0.835	***0.720	***0.645	***0.725

	Other	***0.799	***0.953	***0.860	***0.703	***0.893

Deprivation		0.989	0.999	0.994	***0.910	***1.199

***Practice level predictors***		**Standardised Odds Ratios**				

Practice list size		***0.319	***0.616	***0.407	***0.556	***0.839

Full Time Equivalence ratio per 10,000 patients		***1.268	***1.217	***1.134	0.999	***1.065

Overall reported achievement (comparable indicators only)		***1.202	***1.212	***1.129	***1.120	***1.087

Emergency admissions per 1000 patients		***0.718	***0.883	***0.701	***0.748	0.989

Number of new registrations		***1.237	1.003	***1.144	*1.068	*1.036

Practice Index of Multiple Deprivation score (aggregated from patient sample)		**0.869	***0.862	**0.854	*0.926	***0.929

***Region predictors (practice-level)***		**Standardised Odds Ratios**				

Strategic Health Authority	North East	-	-	-	-	-

	North West	**0.830	1.000	***0.789	*0.893	*0.925

	Yorkshire/Humber	***0.689	0.942	***0.774	***0.839	***0.907

	East Midlands	***0.567	0.972	***0.550	***0.699	***0.854

	West Midlands	***0.600	0.897	***0.700	***0.758	***0.807

	East Of England	***0.572	0.892	***0.594	***0.703	***0.796

	London	***0.434	***0.625	***0.588	***0.607	***0.604

	South East Coast	***0.546	0.983	***0.543	***0.719	***0.698

	South Central	***0.762	0.912	**0.766	**0.846	***0.808

	South West	***0.717	0.961	***0.696	**0.865	***0.881

* = p < 0.05; ** = p < 0.01; *** = p ≤ 0.001

Predictors excluded from the table had no notable (0.86<OR < 1.18) association with any access domain

**Table 4:** Associations between predictors and patient satisfaction and experience, multilevel regression on working respondents only.

		Access domains				
		
		Q2	Q4	Q6	Q8	Q9
		
		Satisfied with getting through on the phone	Able to get appointment same day or next 2 days	Able to get appointment >2 days in advance	Able to get appointment with particular GP	Satisfied with hours GP surgery open
***Number of cases in regressions***		757,687	451,016	337,432	333,649	757,067

***Number of practices in regressions***		8,038	8,037	8,038	8,037	8,038

***Patient level predictors***		**Standardised Odds Ratios**				

Gender	Male	-	-	-	-	-

	Female	***0.951	**1.032	***0.857	***1.047	***0.897

Age group	18 - 34	-	-	-	-	-

	35 - 54	***1.114	***1.044	***1.057	***1.255	***1.044

	55 - 74	***1.169	***1.184	***1.177	***1.523	***1.034

	75+	***1.508	*1.273	***1.731	***2.240	*1.188

Number of appointments (in last 12 months)	0 - 3	-	-	-	-	-

	4 - 6	***0.914	***1.167	***1.212	***1.290	***0.871

	7+	***0.921	***1.403	***1.374	***1.467	***0.960

Parent or legal guardian of any children under 16 currently living in household	No	-	-	-	-	-

	Yes	***0.944	***1.201	***0.962	***0.902	1.008

Employment status	Full-time paid work	-	-	-	-	-

	Part-time paid work	***1.137	***1.261	***1.133	***1.206	***1.430

Travel time to work	Less than 10 min	-	-	-	-	-

	10-30 minutes	*0.978	***0.923	***0.952	**0.954	***0.901

	31 minutes - 1 hour	***0.912	***0.831	***0.888	***0.885	***0.644

	More than 1 hour	***0.813	***0.732	***0.785	***0.750	***0.492

	Live on site	0.958	***0.879	***0.879	0.948	***0.870

Typical working hours†	Weekday office hrs	-	-	-	-	-

	Weekday mornings	***1.139	***1.221	***1.076	1.037	***1.885

	Weekday evenings or afternoons	***1.148	***1.243	*1.049	0.983	***1.899

	Other work pattern	***1.074	***1.118	1.020	1.014	***1.555

	Working hours vary	***1.065	***1.085	0.998	0.998	***1.454

Can take time away from work to visit GP	No	-	-	-	-	-

	Yes	***1.984	***2.047	***1.877	***1.874	***3.209

Ethnicity	White British	-	-	-	-	-

	Other white	***0.851	***0.905	***0.882	***0.837	***0.858

	Black	***0.894	**1.097	***0.896	***0.726	***1.169

	Asian	***0.708	***0.865	***0.723	***0.669	***0.739

	Other	***0.778	0.966	***0.832	***0.739	***0.906

	Rural	*1.033	*0.960	*1.005	**1.023	**0.968

***Practice level predictors***		**Standardised Odds Ratios**				

Practice list size		***0.347	***0.683	***0.424	***0.542	***0.869

Full Time Equivalence ratio per 10 k patients		***1.288	***1.246	***1.146	0.979	***1.057

Overall reported achievement (comparable indicators only)		***1.199	***1.209	***1.116	***1.109	***1.085

Emergency admissions for patients on list per 1000 patients		***0.735	**0.900	***0.712	***0.759	0.989

Standardised Mortality Ratios of people under 65		*0.905	***0.859	*0.910	*0.925	*1.046

Number of new registrations		***1.252	0.981	***1.174	1.055	1.011

Practice Index of Multiple Deprivation score (aggregated from patient sample)		**0.878	**0.877	**0.862	0.950	***0.932

***Region predictors (practice-level)***		**Standardised Odds Ratios**				

Location (Strategic Health Authority)	North East	-	-	-	-	-

	North West	**0.840	1.008	**0.793	*0.871	0.972

	Yorkshire/Humber	***0.709	0.945	**0.778	**0.830	*0.936

	East Midlands	***0.557	0.950	***0.535	***0.663	***0.846

	West Midlands	***0.605	0.894	***0.704	***0.748	***0.839

	East Of England	***0.583	0.899	***0.591	***0.686	***0.836

	London	***0.444	***0.641	***0.630	***0.616	***0.670

	South East Coast	***0.554	0.997	***0.558	***0.695	***0.744

	South Central	***0.757	0.899	**0.777	**0.829	***0.837

	South West	***0.703	0.939	***0.702	**0.818	**0.903

† Two categories of *Typical working hours *have been dropped because of multi-collinearity (*Overnights*, *Weekends*). Categories were small and the information they contained, regarding variation in satisfaction, was also present in other categories which were retained.

* = p < 0.05; ** = p < 0.01; *** = p ≤ 0.001

Predictors excluded from the table had no notable (0.86<OR < 1.18) association with any access domain

**Table 5:** Estimates of between-practice variance and percentage explained by patient- and practice-level predictor variables.

		All respondents		Working respondents only	
		
		Empty model	Full model	Empty model	Full model
Q2. Satisfied with getting through on the phone	Between practice variance Practice variance explained*	1.32 (0.02) -	0.94 (0.02) 29.3%	1.24 (0.02) -	0.88 (0.02) 29.4%

Q4. Able to get appointment same day or next 2 days	Between practice variance Practice variance explained	0.85 (0.02) -	0.68 (0.01) 19.1%	0.84 (0.02) -	0.67 (0.01) 19.9%

Q6. Able to get appointment >2 days in advance	Between practice variance Practice variance explained	1.31 (0.02) -	1.08 (0.02) 17.6%	1.34 (0.03) -	1.13 (0.02) 15.5%

Q8. Able to get appointment with particular GP	Between practice variance Practice variance explained	0.60 (0.01) -	0.44 (0.01) 26.6%	0.64 (0.01) -	0.48 (0.01) 25.0%

Q9. Satisfied with hours GP surgery open	Between practice variance Practice variance explained	0.20 (0.004) -	0.15 (0.003) 23.9%	0.20 (0.004) -	0.14 (0.004) 26.7%

* Using empty model as reference

### Analysis of full sample

Patient age, employment status, and ethnicity were associated with satisfaction and experience in all five domains (Table 3). Satisfaction and positive experience increased with increasing age, were lower amongst those working full-time than in other groups, and were in most instances lower amongst all non-white ethnic groups, most notably patients who described their ethnicity as Asian or mixed-Asian. The presence of long-standing health problems affecting daily activities was associated with reduced ratings in all domains. However, patients who were frequent attendees at their practice reported easier access to appointments. Patient gender only appeared to affect ability to book an appointment in advance.

**Figure 1:**
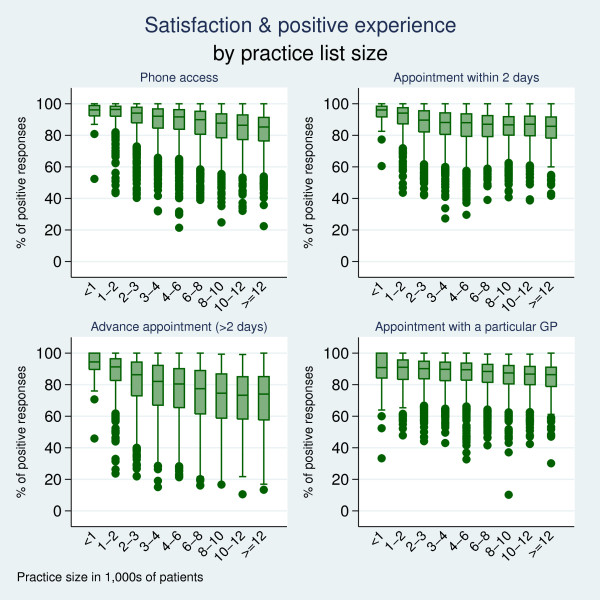
**Satisfaction and positive experience by practice list size**.

Practice size was a strong practice-level predictor, with larger practices receiving poorer ratings on all domains of satisfaction and experience. Figure 1 illustrates that in addition to having higher mean ratings, between-practice variability in access was also lower in smaller practices for most domains. For example, the standard deviations for satisfaction on getting through on the phone were: 23.8% for small (< 2,000 patients) and 37.6% for large practices (> 10,000 patients). Practices with a higher GP-to-patient ratio were reported as being better in terms of phone access and the availability of appointments within the next two days. Patients also found it more difficult to make appointments in practices serving more deprived populations.

Two variables potentially measuring aspects of quality of care were also associated with scores on this questionnaire. Patient ratings were higher for practices with higher scores on the Quality and Outcomes Framework, and lower for those with higher rates of emergency admission. Relative to an 80% baseline, we estimate from the regression results that an increase in QOF reported achievement of 10 points was associated with an increase in satisfaction and experience rating of up to 3.4% (eg getting through on the phone: 3.2%, urgent appointment: 3.4%, advance appointment: 2.1%, appointment with a particular doctor: 2.0%).

The only regional predictor of note was location, as defined by Strategic Health Authority. On most domains, patients in the North East reported the higher levels of satisfaction/experience, while those in London reported the lowest.

The patient- and practice-level predictor variables used in the regression models explained quite sizable percentages of the variability between practices (Table 5). In the all-respondents regressions the percentage ranged from 17.6% in the case of advance appointments to 29.3% for getting through on the phone. For the working-respondents regressions percentages varied from 15.5% to 29.4%.

### Analysis of patients in full- or part-time employment

For patients in employment, satisfaction and positive experience in all domains was substantially higher amongst patients able to take time off work to visit their GP. Patients who had a commute of more than one hour to work were more dissatisfied on all domains than those with short commutes. Compared to part-time workers, people in full-time employment rated access as poorer on most domains. Those working normal office hours found it harder to get an immediate appointment and were less satisfied with practice opening hours than people with other working patterns. The raw means in table 2 suggest that working people in part-time employment, or with short commutes, or allowed time off to attend the practice, rated the access domains in a similar way to most of the non-working groups.

### Analysis of patient- and practice-level interactions

We conducted further analyses to examine whether individual patient responses were related to the distribution of these characteristics in the general population local to each practice (i.e. area effects over and above individual patient effects). To examine this question we repeated the multi-level regressions adding in three interaction terms between patient and practice-population variables: patient ethnicity (white v non-white) by the percentage of whites in the practice population; patient age by percentage in the population under 45; patient working full-time by percentage in the population working full-time. These analyses controlled for differences in all other measured factors.

Table 6 summarises the results. After controlling for all other factors, area characteristics had a number of sizable impacts on satisfaction over and above their effects at the individual patient level. The size of the area non-white population substantially reduced satisfaction and experience ratings amongst both white and non-whites on all five domains, but with a slightly greater effect amongst non-white patients themselves. Ratings of phone access, the ability to obtain an advance appointment and opening hours by both young (under 45 years) and older people were marginally higher in areas with a mainly young population. Rating of phone access, the ability to obtain an early appointment, and opening hours, were lower in areas of high full-time employment, both for people working full-time and for those not. Inclusion of these interaction terms increased the between-practice variance in access ratings explained by the model by between one and two percent.

**Table 6:** Summary of patient- and practice-level interactions, from multilevel regressions on all respondents.

Patient characteristic		Not White		White		Under 45 yrs (18-44)		45 yrs plus		Not working Full-time		Working full-time	
Practice population^1^		20% White	80% White	20% White	80% White	20% < 45	80% < 45	20% < 45	80% < 45	20% FT	80% FT	20% FT	80% FT

Q2: Satisfied with getting through on the phone	% Satisfied	72.7%^3^	87.4%	76.6%	89.4%	85.2%	89.1%	89.8%	92.6%	92.4%	88.2%	89.8%	84.5%
	
	Effects^2^	White patient: 1.076*** % Population White: 1.337*** Interaction: 0.955***				Patient aged under 45: 0.912*** % Population aged <45: 1.072*** Interaction: 0.946***				Patient working FT: 0.831*** % Population working FT: 0.932*** Interaction: 1.003 ns			

Q4: Able to get appointment same day or next 2 days	% Satisfied	77.9%	85.8%	80.4%	87.6%	85.0%	85.1%	89.5%	89.5%	90.9%	88.5%	85.2%	81.6%
	
	Effects	White patient: 1.013** % Population White: 1.175** Interaction: 0.976***				Patient aged under 45: 0.893*** % Population aged <45: 1.000 ns Interaction: 0.971***				Patient working FT: 0.772*** % Population working FT: 0.963** Interaction: 0.988 ns			

Q6: Able to get appointment >2 days in advance	% Satisfied	64.4%	76.4%	67.4%	78.6%	72.5%	76.5%	80.2%	83.4%	83.0%	79.8%	76.6%	72.6%
	
	Effects	White patient: 1.056*** % Population White: 1.196*** Interaction: 0.946 ns				Patient aged under 45: 0.907*** % Population aged <45: 1.042 * Interaction: 0.995 ns				Patient working FT: 0.826*** % Population working FT: 0.969 ns Interaction: 1.001 ns			

Q8: Able to get appointment with particular GP	% Satisfied	75.7%	83.3%	83.0%	88.6%	82.9%	84.1%	90.0%	90.7%	90.5%	89.4%	85.3%	83.8%
	
	Effects	White patient: 1.129*** % Population White: 1.153*** Interaction: 0.952**				Patient aged under 45: 0.841*** % Population aged <45: 1.016 ns Interaction: 1.011 ns				Patient working FT: 0.816*** % Population working FT: 0.984 ns Interaction: 0.971***			

Q9: Satisfied with hours GP surgery open	% Satisfied	72.9%	79.9%	75.9%	82.4%	75.2%	79.8%	83.5%	86.9%	88.0%	86.5%	75.9%	73.2%
	
	Effects	White patient: 1.043*** % Population White: 1.125*** Interaction: 0.989*				Patient aged under 45: 0.908*** % Population aged <45: 1.052*** Interaction: 0.904***				Patient working FT: 0.654*** % Population working FT: 0.981 ** Interaction: 0.992 ns			

^1^Population-level characteristics were used in continuous form in the regression analysis, but for descriptive purposes, results are presented for population percentages of 20% and 80%

^2 ^Odd ratios from multi-level logistic regression on all respondents.

^3^Estimated percentage from the regression model, controlling for all other factors in the model.

* = p < 0.05; ** = p < 0.01; *** = p ≤ 0.001

## Discussion and Conclusions

### Strengths and limitations

99% of practices in England were included in the survey resulting in a very large sample with almost two million respondents. The overall response rate to the survey was low (40.6%), and so results could have been affected by response bias. While previous research in patient satisfaction and experience with access suggests that non response is commoner among men,^18 ^it is unlikely that any over-representation of females in the present case will have introduced bias since the effect of gender was estimated to be very small. It is also probable that non-respondents tended to be younger (mean practice patient age in the sample was 53.8 and in the 2001 census it was 47.3). Since younger patients tend to be more negative in their responses, satisfaction and positive experience with access might have been overestimated. However, a recent study of a later but similar questionnaire suggested that that response bias in practice estimates of access to care was small and not consistent in direction across individual questions in the survey [17]. The representativeness of the total sample would also have been affected by the survey sampling design which, so as to obtain a minimum number of responses from all practices, relative to list size oversampled patients from smaller practices.

Nonrepresentativeness may lead to bias in subgroup scores (Tables 1 and 2) as these are calculated without weighting for sampling fractions and non-response, but is less of an issue for the estimation of the strength of relationships between variables, in this case the odds-ratios from the multi-level logistic regressions.

The analyses identify patient and practice characteristics that explain quite substantial percentages - up to 30% - of the variation in practice access ratings. Patient demographic factors with the greatest impact on satisfaction/experience, across all domains of access, were age (older people more satisfied), ethnicity (White British most satisfied, Asians least satisfied), and employment status (full-time employed least satisfied, retired people most satisfied). Amongst those in employment, we found that being not being able to take time off work to visit the GP was a key factor in determining responses across all domains. Other factors that freed up time, such as working part-time or having a short commute, were associated with more positive responses to the questionnaire. Despite substantial variation in reported practice opening hours, we found no notable relationship between total hours of availability and responses to any of the access questions - including satisfaction with opening hours themselves. This result held even among the working population.

Practice size emerged from the analysis as the dominant practice-level factor influencing experience of access. Patients in small practices were generally reported easier access than patients in larger practices. Small practices were also less variable in terms of the access they provided. Satisfaction with telephone access was particularly increased in smaller practices. It may be that smaller practices can maintain a better ratio of telephone lines/administrative staff to volume of calls. This finding is consistent with previous studies, in which smaller practices were associated with high patient ratings of access and continuity of care [18-20]. Using the estimated coefficients from the full-sample regression analysis and a baseline level of 80%, the practice size effect on satisfaction and experience can be expressed in linear terms: a practice list size increase of 1,000 was associated with a reduction in experience and satisfaction of up to 2.4% (reductions relating to getting through on the phone: 2.4%, urgent appointment: 1.0%, advance appointment: 1.9%, and appointment with a particular doctor: 1.2%). As practices in the UK are tending to become larger, consideration needs to be given to how the potential benefits associated with small practice size can be retained.

We found that patient ethnic identity affected reported satisfaction and experience on all domains of access. Many factors are known to influence the way in which different patient groups rate their care, including differences in health needs, expectations, and response tendencies, as well as experience per se [21-23]. Some research suggests that expectations are different in some ethnic minorities, even when experience is similar [21]. However, we further found that the ethnic make-up of the area population had an impact on satisfaction/experience over and above a patient's own ethnic identity. In particular, the larger the area non-white community, the more likely that both white and non-white patients were to give lower ratings on all domains of access after controlling for other patient and area characteristics. Comparing areas with small (20%) and large (80%) white populations, the area effect was consistently stronger than the association with ethnicity of individual respondents. The area effect was also slightly greater amongst non-white patients. It is not obvious why there exists such a strong area effect, across both white and non-white patients, particularly once other factors such as area deprivation have been controlled for. It may be that individual expectations of care and rating tendencies are modified by the dominant views within the wider local community.

These findings are broadly consistent with other literature on patient evaluations of their care. Studies in both the UK and in other countries have found that younger patients, patients from ethnic minority groups and patients living in socio-economically deprived localities tend to have less favourable evaluations of their care compared to older, white or affluent populations [24,25]. These differences could be due to differences in actual care received or to different response tendencies of individual population groups. If the differences are due to differences in care received, then the results can be used to identify areas where quality improvement should be focused. However, differences in response tendency of different population groups could be used as an argument for case-mix adjustment when comparing the results for practices serving different populations [26]. Our finding of a strong area-ethnicity effect suggests that case-mix adjustment for ethnicity would need to consider area as well as patient characteristics in this respect, if it is to be at all accurate.

Satisfaction and experience on some domains also appeared to be related to aspects of the quality of care provided by the practice - Quality and Outcomes Framework clinical indicator scores and rates of emergency admission. Previous research has not always found consistent relationships between access to primary care and rates of preventable hospital admission [27,28]. Our finding raises the possibility of a causal link between difficulty getting appointments and emergency admissions. This result merits further investigation, in particular whether availability of care in normal working hours influences demand for care out of hours, which is a time when many emergency admissions occur. Overall, the results of this study suggest a number of areas where responses to survey questions on access suggest areas for potential quality improvement.

## Competing interests

The authors declare that they have no competing interests.

## Authors' contributions

All authors were involved in conceptualizing and planning the study. EK and DR decided on the analyses and EK carried them out. EK drafted the manuscript and MR and DR critically revised it. All authors contributed to the interpretation of the results. All authors read and approved the final manuscript.

## Pre-publication history

The pre-publication history for this paper can be accessed here:

http://www.biomedcentral.com/1471-2296/11/61/prepub

## Supplementary Material

Additional file 1 **2008 GP patient survey questionnaire**. Click here for file
