# Supplementary material for: Patient experience of access to primary care: identification of predictors in a national patient survey
Source: BMC Fam Pract. 2010 Aug 28;11:61. doi: 10.1186/1471-2296-11-61 (PMC2936332; doi:10.1186/1471-2296-11-61)
Supplement: Additional file 1 — 2008 GP patient survey questionnaire. [file 1471-2296-11-61-S1.PDF]

Additional file 1

2008 GP patient survey questionnaire		
	**Question**	**Answers**
**Section A: Getting to see the doctor**		
Q1	When was the last time you saw a doctor at your GP surgery?	☐ Less than 3 months ago<br>☐ Between 3 and 6 months ago<br>☐ More than 6 months ago
Q2	In general, are you satisfied with how easy it is to get through to someone on the phone at your doctor's surgery?	☐ Yes<br>☐ No
Q3	In the last 6 months, have you tried to get an appointment with a doctor fairly quickly about any matter?	☐ Yes<br>☐ No
Q4*	Think about the last time you tried to get an appointment with a doctor fairly quickly**: Were you able to get the appointment on the same day or on the next 2 days the surgery was open?	☐ Yes<br>☐ No
Q5	In the last 6 months, have you wanted to book ahead[†] for an appointment with a doctor?	☐ Yes<br>☐ No
Q6*	Last time you wanted to, were you able to get an appointment with a doctor more than 2 full days in advance?	☐ Yes<br>☐ No
Q7	In the last 6 months, have you ever wanted to make an appointment with a particular doctor at your GP surgery?	☐ Yes<br>☐ No
Q8*	Last time you wanted to, were you able to make an appointment with a particular doctor - even if it meant waiting longer?	☐ Yes<br>☐ No
Q9	Over the last 6 months or so, were you satisfied with the hours your GP surgery was open?	☐ Yes<br>☐ No
Q10*	I was dissatisfied because…	☐ the surgery was not open early enough in the morning<br>☐ the surgery was not open around lunchtime<br>☐ the surgery was not open late enough in the evening<br>☐ the surgery was not open on a Saturday<br>☐ the surgery was not open on a Sunday<br>☐ of some other reason
**Section B: Referrals to hospital**		
Q11	In the last 6 months, has your doctor referred you to see a specialist?	☐ Yes<br>☐ No
Q12*	Think about the last time your doctor referred you to see a specialist. Did your doctor talk with you about a choice of hospital for your appointment?	☐ Yes<br>☐ No
**Section C: Some questions about you**		
Q13	Are you male or female?	☐ Male<br>☐ Female

**2008 GP patient survey questionnaire**		
	**Question**	**Answers**
Q14	How old are you?	☐ Under 18<br>☐ 18-19<br>☐ 20-24<br>☐ 25-34<br>☐ 35-44<br>☐ 45-54<br>☐ 55-59<br>☐ 60-64<br>☐ 65-74<br>☐ 75-84<br>☐ 85 or over
Q15	How many appointments have you made with a doctor at your GP surgery in the last 12 months?	☐ None<br>☐ 1<br>☐ 2<br>☐ 3<br>☐ 4<br>☐ 5<br>☐ 6<br>☐ 7-10<br>☐ 11-15<br>☐ 16-25<br>☐ 26 or more
Q16	Are you a parent or legal guardian for any children aged under 16 currently living in your household?	☐ Yes<br>☐ No
Q17	Which of these best describes what you are doing at present?<br>*If more than one of these applies to you, please tick the main ONE*	☐ Full-time paid work (30 hours plus per week)<br>☐ Part-time paid work (under 30 hours per week)<br>☐ Full-time education at school , college or university<br>☐ Unemployed<br>☐ Permanently sick/disabled<br>☐ Looking after home<br>☐ Doing something else
Q18*	In general how long does your journey take from home to work (door to door)?	☐ Less than 10 minutes<br>☐ 10-30 minutes<br>☐ 31 minutes – 1 hour<br>☐ More than 1 hour<br>☐ Live on site
Q19*	Which of the following most reflects your typical working hours?<br>*If more than one of these applies to you, please tick the main ONE*	☐ Weekday office hours (starting around 8 or 9am and finishing around 5 or 6 pm)<br>☐ Weekday mornings<br>☐ Weekday afternoons/evenings<br>☐ Overnights<br>☐ Weekends<br>☐ Other work pattern<br>☐ Working hours vary
Q20	If you need to see a doctor at your GP surgery during your typical working hours, can you take time away from your work to do this?	☐ Yes<br>☐ No
Q21	Do you have any of the following long-standing conditions?<br>*Include problems which are due to old age.*<br>*Please tick all boxes which apply.*	☐ Deafness or severe hearing impairment<br>☐ Blindness or severe visual impairment<br>☐ A condition that substantially limits one or more basic physical activities such as walking, climbing stairs, lifting or carrying<br>☐ A learning difficulty<br>☐ A long-standing psychological or

2008 GP patient survey questionnaire		
	**Question**	**Answers**
		emotional condition<br>☐ Other, including any long-standing illness<br>☐ No, I do not have a long-standing condition
Q22	Does your long-standing health problem or disability mean you have substantial difficulties doing day-to-day activities?<br>*Include problems which are due to old age.*	☐ Yes<br>☐ No
Q23	Do you have carer responsibilities for anyone in your household with a long-standing health problem or disability?	☐ Yes<br>☐ No
Q24	What is your ethnic group?	A. White<br>☐ British<br>☐ Irish<br>☐ Other White (write in)<br><br>B. Mixed<br>☐ White and Black Caribbean<br>☐ White and Black African<br>☐ White and Asian<br>☐ Other Mixed (write in)<br><br>C. Asian or Asian British<br>☐ Indian<br>☐ Pakistani<br>☐ Bangladeshi<br>☐ Other Asian (write in)<br><br>D. Black or Black British<br>☐ Caribbean<br>☐ African<br>☐ Other Black (write in)<br><br>E. Chinese or other ethnic group<br>☐ Chinese<br>☐ Other (write in)
* These questions do not apply to all respondents<br>** ’Fairly quickly’ means on the same day or the next two days the surgery was open<br>† ‘Book ahead’ means booking an appointment more than 2 full days in advance		
